# Subthreshold Exudative Choroidal Neovascularization (CNV): Presentation of This Uncommon Subtype and Other CNVs in Age-Related Macular Degeneration (AMD)

**DOI:** 10.3390/jcm11082083

**Published:** 2022-04-07

**Authors:** Vivian Paraskevi Douglas, Itika Garg, Konstantinos A. A. Douglas, John B. Miller

**Affiliations:** 1 Harvard Retinal Imaging Lab, Massachusetts Eye and Ear, Harvard Medical School, 243 Charles St., Boston, MA 02114, USA; douglasvivianp@gmail.com (V.P.D.); itika_garg@meei.harvard.edu (I.G.); douglaskonstantinos@gmail.com (K.A.A.D.); 2 Retina Service, Massachusetts Eye and Ear, Department of Ophthalmology, Harvard Medical School, 243 Charles St., Boston, MA 02114, USA

**Keywords:** subthreshold exudative choroidal neovascularization, CNV, age-related macular degeneration, AMD, occult CNV, classic CNV, retinal angiomatous proliferation, mixed CNV, nonexudative CNV, quiescent, macular neovascularization, MNV

## Abstract

Age-related macular degeneration (AMD) is a leading cause of irreversible vision loss in people over the age of 50 worldwide. Exudative or neovascular AMD is a more severe subset of AMD which is characterized by the presence of choroidal neovascularization (CNV). Recent advancements in multimodal ophthalmic imaging, including optical coherence tomography (OCT) and OCT-angiography (OCT-A), have facilitated the detection and characterization of previously undetectable neovascular lesions and have enabled a more refined classification of CNV in exudative as well as nonexudative AMD patients. Subthreshold exudative CNV is a novel subtype of exudative AMD that typically presents asymptomatically with good visual acuity and is characterized by stable persistent or intermittent subretinal fluid (SRF). This review aims to provide an overview of the clinical as well as multimodal imaging characteristics of CNV in AMD, including this new clinical phenotype, and propose effective approaches for management.

## 1. Introduction

Age-related macular degeneration (AMD) is a leading cause of irreversible vision loss in people over the age of 50 worldwide [1]. By 2040, AMD is projected to affect nearly 288 million people worldwide (95% CI, 205–399) [2]. The World Health Organization has listed AMD as a “Priority eye disease” because of its increasing prevalence among the geriatric population and the paltry treatment options available. Indeed, further research aimed at the development of novel diagnostic, therapeutic, and preventive approaches is needed [3].

The clinical hallmark of AMD is the presence of drusen, which are focal deposits of acellular debris. These are usually found bilaterally and are located between the retinal pigment epithelium (RPE) and Bruch’s membrane (BM) [1]. AMD can be classified as either nonexudative (dry) or exudative (wet). Nonexudative AMD is the most common type, affecting 75–85% of AMD patients, and is further divided into early, intermediate, and advanced stages based on the number and size of the drusen and the degree of pigmentary changes and geographic atrophy (GA) [4].

Exudative AMD occurs secondary to choroidal neovascularization (CNV) and can present in fluorescein angiography (FA) as leakage that is the release of fluid and serum components due to blood–ocular barrier disruption. On optical coherence tomography (OCT), intraretinal fluid (IRF) and subretinal fluid (SRF) accumulation are also seen which are the direct result of decreased ability of the tissue to remove the excess fluid [5]. Lipid/hard exudates indicate the presence of a chronic process and leakage and are better detected on OCT, as in some cases these are too small to be visible by fundus examination [5]. Hemorrhages can be also found in exudative AMD and can be seen at different layers (sub-RPE, intraretinal, preretinal, subretinal); thus, OCT is an important imaging tool in their accurate detection. Moreover, subretinal hyperreflective exudative material (SHRM) is another common feature in patients with exudative AMD and is also considered an OCT biomarker of fibrosis. SHRM is typically composed of serum, fibrin, and inflammatory cells and is associated with poor prognosis [5,6]. 

Exudative AMD is an advanced form of AMD involving the abnormal growth of blood vessels that originate from the choroid and project into the outer retinal layer. However, it can also arise from the retinal circulation (type 3 neovascularization) [7]. As a result, it has been suggested that the term “macular neovascularization (MNV)” should replace CNV [7]. Although the exudative AMD affects approximately 10–15% of individuals with AMD, it accounts for more than 80% of cases with severe visual loss or legal blindness [8]. Interestingly, Bacci et al., recently described a novel clinical phenotype, the exudative non-neovascular AMD, using clinical and multimodal imaging where three patients (three eyes) with dry AMD presented with IRF without evidence of neovascularization or alterations in retinal vasculature [9].

The use of fluorescein and indocyanine green dyes has improved our ability to visualize the retinal and choroidal vasculature. They help detect and characterize the different types of MNV (Table 1) and also enhance our understanding of their pathophysiology [10,11]. Optical coherence tomography–angiography (OCT-A), a functional extension of OCT, is a dye-less, non-invasive imaging modality that employs motion contrast imaging of blood flow to generate high-resolution volumetric angiographic images [12]. Its depth-resolved nature provides unique advantages in the investigation of different types of MNV and the identification of previously undetectable neovascular lesions. OCT-A has contributed to early detection, timely management, monitoring of progression, and response to treatment of MNV in both exudative and nonexudative AMD patients.

This review aims to provide an overview of the types of CNV/MNV with a primary focus on subthreshold CNV, a new and uncommon subtype of exudative AMD. We discuss its unique clinical and multimodal imaging characteristics and present the effective management approaches that have been proposed thus far for the same.

## 2. Exudative AMD

Macular neovascularization develops in exudative AMD due to age-related, genetic, and environmental factors [13]. Age-related alterations in the outer retinal layer include the deposition of insoluble material, increased BM thickening, and choriocapillaris thinning. This can lead to photoreceptor or RPE hypoxia, which can in turn stimulate VEGF release favoring angiogenesis [14]. Determining the patterns of these newly formed neovascular lesions and their relation to clinical activity is important for optimizing patient outcomes.

In 1991, the first system to classify exudative AMD was developed for the Macular Photocoagulation Study (MPS). The classifications were built based on the FA leakage patterns, classic vs. occult leakage [15]. In 1994, Gass et al., described two histologic subtypes of CNV; type 1, where the CNV is located below the RPE and is composed of fibrovascular tissue at the choroidal side of the RPE, and type 2, where the CNV is located above the RPE and mainly composed of subretinal fibrovascular tissue [16,17]. A combined subtype of CNV with features of both type 1 and 2 was also reported in the same study [16]. 

## 3. Type 1 MNV

Type 1 MNV (old term: type 1 CNV or occult CNV) is the most common variant of exudative AMD and occurs in 60–85% of patients [18,19]. The neovascular complexes arise from the choriocapillaris, and after penetrating through BM they reside within the sub-RPE space [9,20]. The neovascularization accompanying this subtype is usually slowly progressive with patients having overall better visual outcomes at 5-year follow-up compared to other CNV types but similar visual acuity (VA) change [21]. Type 1 CNV can be evaluated using fundus photography, FA, ICGA, OCT, and OCT-A.

Fundus photography of patients with exudative AMD and type 1 CNV reveals non-specific signs such as drusen and pigment mottling. On FA, the new vessels are usually described as poorly defined or occult CNV. Other FA findings are stippled hyper-fluorescence, late leakage of undetermined source, vascularized RPE, and pigment epithelial detachment (PED) [15,22]. On ICGA, a well or poorly defined plaque (hyperfluorescent area) or focal and ill-defined CNVs can be seen in approximately 60%, 30%, and 10% of patients, respectively [23]. Pece et al., concluded that well-defined plaques could enlarge up to 40% within one year, but without significant visual loss [23]. On OCT, type 1 CNV appears as RPE elevation with heterogeneous reflectivity. Interestingly, on OCT-A, the type 1 CNV appears smaller than the leakage in conventional FA [24]. Topographic ICGA has shown to be a more reliable method in identifying this vascular complex, as the conventional ICGA can often underestimate its actual size due to overlying masking phenomena [24]. On OCT-A, the abnormal vessels appear below the RPE layer with different morphological patterns being described, but without having a significant clinical correlation to its activity [25]. More specifically, in up to 75% of cases a large and highly organized neovascular complex is seen and vessels can either branch off the center of the lesion (“medusa” shape) or radiate from one side (“seafan” shape) [25].

Intravitreal injections of anti-vascular endothelial growth factor (anti-VEGF) have been considered the gold standard for the management of CNV secondary to AMD but also secondary to other macular diseases where currently four medications have been widely and effectively used in clinical practice: ranibizumab, bevacizumab, aflibercept, and brolucizumab [26]. Patients typically receive three monthly injections and later can follow a pro re nata (PRN) (injection in active disease), treat-and-extend (T&E) (modifying the interval of injections), or fixed (2 monthly injections within the first 12 months irrespective of the level of disease activity) regimen [27]. 

## 4. Type 2 MNV

Type 2 MNV (old term: type 2 CNV or classic CNV) is a relatively uncommon and more aggressive subtype of CNV where the complexes arise from the choroid, traverse BM and the RPE monolayer, and then spread to the subretinal space [8]. It occurs in 9% of patients diagnosed with exudative AMD and usually presents with acute visual symptoms [20]. Patients with type 2 CNV commonly exhibit synchronous reticular pseudodrusen and thin subfoveal choroids [28].

Similar to type 1 CNV, there are non-specific findings on fundus examination with gray-white subretinal changes, retinal edema, hard exudates, and subretinal and intraretinal hemorrhages commonly seen. On FA, early hyper-fluorescence and late pooling in the subretinal space are noted. In addition, the vascular complexes are usually well-defined. Due to the intense hyper-fluorescence of the choroidal circulation, it is often difficult to detect the vessels on ICGA [22]. On SD-OCT, the lesion is localized above the RPE and below the photoreceptor outer segments, while disruption of inner/outer segment photoreceptor junction, subretinal fluid, hyperreflective exudation in the subretinal space, and the presence of intraretinal cysts are usually seen [29].

Management of type 2 MNV is similar to type 1 MNV, with patients following monthly injections for the first three months while the treatment regimen is modified in the follow-up (fixed, PRN, T&E) [27].

## 5. Type 3 MNV

Type 3 MNV [old term: retinal angiomatous proliferation (RAP)] is a distinct form of exudative AMD characterized by neovascularization rising from the deep capillary plexus and growing toward the outer retina. It is classified into three stages based on the origin and progression of the neovascular changes; intraretinal (stage 1), subretinal (stage 2), and choroidal (stage 3) neovascularization [30]. It can be seen in up to approximately 20% of exudative AMD and most commonly in older patients compared to other forms of exudative AMD [31]. Histopathological studies have shown that the complexes grow out of the neuroretina into the subretinal space [32]. In addition, there is expression of VEGF in the neovascular complexes as well as of hypoxia inducible factors-1 alpha and hypoxia inducible factor-2 alpha in macrophages and neovascular endothelial cells [33].

In the study by Jung et al., 39.9% of exudative AMD cases were Type 1, 9.0% were Type 2, and 16.9% were mixed, of which 80% were Type 1 and 2 followed by 15.5% of mixed Type 1 and 3 (retinal angiomatous proliferation, RAP) and 4.4% of mixed type 2 and 3 [20].

Management approaches of type 3 MNV are essentially the same as in type 1 and type 2 MNV [8]. Interestingly, in the recent study by Sharma et al. it was found that the presence of only intraretinal fluid was associated with better visual outcomes, need of fewer injections, and less subfoveal atrophy compared to other exudative AMD types [34].

## 6. Atypical Types of CNV (Identified by OCT-A)

### Subthreshold Exudative CNV 

Subthreshold CNV comprises an uncommon subtype of exudative CNV which was recently reported by Roh et al. [35]. In this retrospective study, they reviewed 3773 patient records from the past two decades with exudative AMD according to the Age-Related Eye Disease Study 2 and having a VA equal to or greater than 20/25. They included patients with (i) a follow-up of at least one year without receiving anti-VEGF injections, (ii) presence of drusen within the macula on fundus examination with or without pigmentary changes, and (iii) irregular RPE elevation on OCT with no or minimal presence of SRF. Eight eyes of six patients met the above-mentioned criteria. A multimodal imaging protocol was applied that included FA, ICGA, OCT, and OCT-A.

The baseline patient demographics comprised 66.7% females (*n* = 4), a mean age of 68 years (range: 58–80 years), and 100% Caucasian (*n* = 6). VA at presentation ranged between 20/20 and 20/25, while the final VA ranged between 20/20 and 20/32 despite the variation in total follow-up time among patients. Though none of the patients were treated throughout this period, 3 of 8 eyes had already received anti-VEGF injections, which could have influenced the growth pattern of the CNV.

**Figure 1:**
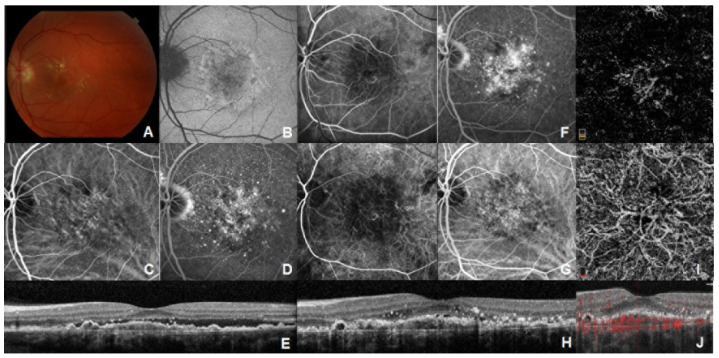
Multimodal imaging of one patient with subthreshold CNV. (**A**) Fundus photography of the left eye with soft drusen and pigmentary changes. Visual acuity (VA) is 20/25. (**B**) Diffuse ring of hyperautofluorescence is noted in the macula. (**C**) ICGA does not demonstrate a clear hot spot or plaque. (**D**) Late phase FA shows diffuse staining corresponding to RPE atrophy and drusen. (**E**) SD-OCT demonstrates irregular RPE elevation with overlying SRF. Fifteen months later, VA decreased to 20/70. (**F**) Early (**left**) and late (**right**) frames of FA demonstrate diffuse leakage of undetermined source in the left eye. (**G**) ICGA reveals a hypercyanascent neovascular membrane in both early (**left**) and late (**right**) phase. (**H**) SD-OCT demonstrates a fibrovascular pigment epithelial detachment (PED) with overlying hyperreflective spots and sliver of SRF. (**I**) Corresponding OCT-A of the outer retina (**top**) and choriocapillaris (**bottom**) layer shows a vascular network. (**J**) B scan on choriocapillaris layer reveals a high flow vascular network. Reprinted with permission from ref. [35]. Copyright 2020, SAGE publishing.

**Figure 2:**
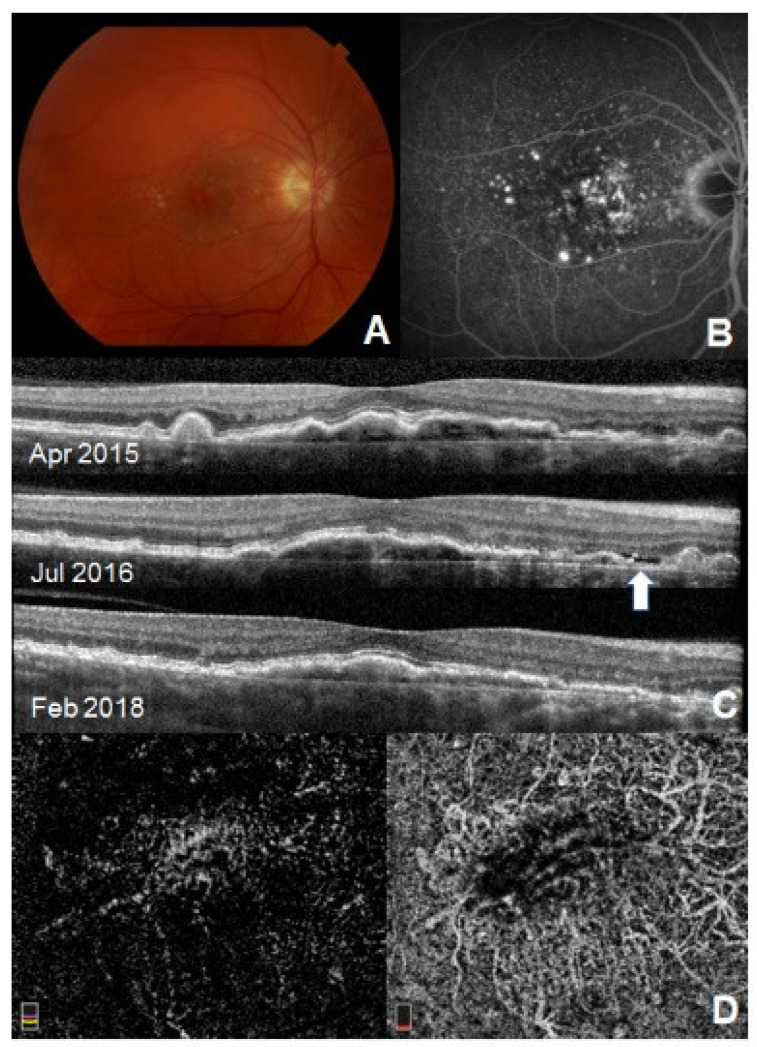
Multimodal imaging of the right eye of the same patient as in Figure 1. Visual acuity is 20/20. (**A**) Fundus photography of the right eye shows pigmentary changes with drusen in macula. (**B**) Late-phase FA shows staining without active fluorescent leakage. (**C**) SD-OCT reveals irregular elevated RPE elevation with moderately reflective material in the sub-RPE space with fluctuating subretinal fluid over the follow-up period (Arrow, SRF) (**D**) OCT-A segmented on the outer retina (**left**) and choriocapillaris (**right**) layer showing vascular network under fovea suggestive of ‘quiescent CNV’. Reprinted with permission from ref. [35]. Copyright 2020, SAGE publishing.

Spectral-domain OCT demonstrated irregular RPE elevation with either stable or intermittent SRF in all eyes. On FA, stippled hyper-fluorescence with late staining but no leakage was noted. In addition, ICGA studies were performed in seven eyes and no definite neovascular complexes or hot spots were identified. However, only two of seven patients showed plaques on ICGA. On OCT-A, 3 mm × 3 mm scans centered on the fovea and vascular networks of variable shape and border were observed in the fovea or perifoveal areas, which were suggestive of type 1 CNV (Figure 1 and Figure 2). Based on clinical and imaging findings, the group decided not to treat these patients but rather to encourage close observation and self-monitoring with frequent follow-up care.

This study proposed that asymptomatic AMD patients with presence of subthreshold exudative CNV and VA of 20/25 or better can be managed with close observation without anti-VEGF treatment and remain stable for long-term follow-up. 

## 7. Nonexudative CNV (CNV in Dry AMD, Subclinical CNV, Quiescent CNV)

Νon-exudative CNV is defined by the presence of CNV when assessed by OCT-A and the absence of hemorrhage or fluid on clinical examination and leakage on angiographic studies. This is usually an incidental finding in asymptomatic patients with dry AMD during routine follow-up visits and is often associated with better visual prognosis. When early or intermediate AMD is associated with extrafoveal RPE atrophy, advanced age, or fellow eye with exudative CNV on OCT-A, it is considered high risk for progression to nonexudative, subclinical AMD [36]. The two-year incidence of conversion to exudative CNV is estimated to be 34.5%, and it is 13.6 times greater when compared to eyes without subclinical CNV [37].

In 2013, Querques et al. first described the term treatment-naïve (quiescent) CNV in patients with intermediate AMD. They described the morphological and functional characteristics of this new entity by means of multimodal imaging [38]. Quiescent CNVs are defined as angiographically detectable CNVs lacking intraretinal/subretinal exudation on repeated OCT for at least six months. On FA, quiescent CNVs appear as type 1 CNV lesions characterized by late speckled hyper-fluorescence and ill-defined borders without leakage or pooling. On mid-late-phase ICGA they appear as hyperfluorescent plaques. Irregularly elevated RPE without presence of fluid is typically present on spectral-domain OCT (SD-OCT) [38].

Carnevali et al. used OCT-A to investigate the features of quiescent CNV in intermediate AMD and for estimating its detection rate using this novel, non-invasive technique [39]. They concluded that quiescent CNV could be assessed and characterized based on its shape (circular or irregular), its margin (well or poorly defined), its location (foveal- involving or sparing), and whether its core (vessel of greater caliber from which smaller vessels branch off) is central, eccentric, or not visible. This resulted in a sensitivity and specificity of 81.8% and 100%, respectively [39].

Furthermore, studies have shown that the presence of the double-layer sign (irregular elevation of the RPE from the underlying intact BM) [40] on structural OCT could also predict the presence of subclinical quiescent neovascularization and may function as a screening tool for nonexudative AMD [41,42]. However, this sign can also be a feature of the pachychoroid disease spectrum such as pachychoroid neovasculopathy (PCN). Hence, polypoidal choroidal vasculopathy (PCV) should be always included in the differential diagnosis [43].

With regard to the treatment of subclinical CNVs, most clinicians have recommended closer observation with more frequent clinic visits for these patients [40,41,42,43,44].

## 8. Discussion

Age-related macular degeneration is the leading cause of blindness worldwide. Given the absence of prevention and the absence of effective treatment strategies for both dry and wet variants, its prevalence is estimated to reach epidemic proportions in the near future [2]. Intravitreal anti-VEGF therapy is considered one of the monumental scientific discoveries in ophthalmology and has since become the gold standard for the management of exudative AMD. The introduction of OCT-A has enabled the detection and characterization of previously unknown subtypes of abnormal vascular complexes in both dry and wet AMD. This has substantially helped us identify new entities and increase our understanding of numerous retinal and choroidal diseases and their diverse presentations. Refining the classification systems and using meaningful and precise language is critical for exploring new and optimizing current treatment strategies. 

In 2016, Carnevali et al. suggested that the term “exudative AMD” should be used to describe CNV with signs of active disease while “neovascular” should be used to refer to quiescent CNV without exudation [39]. The most recent classification system for reporting neovascular AMD is the one proposed by the Consensus on Neovascular AMD Nomenclature (CONAN) group; this could serve as a general overall framework that would likely be revised as new studies are published [5]. In fact, around the same time as the CONAN, the study of Roh et al. was also published [35]. In 2011, Kloos et al. used the term “secondary sick RPE syndrome” to describe the presence of isolated SRF that is refractive to repeated anti-VEGF injections in a subset of patients with exudative AMD [45]. In the study of Roh et al. [35], OCT-A assisted in the identification of neovascularization present underneath the RPE of patients with exudative AMD and intermittent or persistent SRF. Hence, the term “subthreshold exudative CNV” could now be used to describe the cases previously referred to as sick RPE syndrome.

The introduction of intravitreal anti-VEGF in clinical practice has played a key role in the management of exudative neovascular AMD and has led to improved overall visual prognosis. While the presence of subretinal fluid often prompts the initiation of intravitreal injections, there are some cases where treatment can be deferred as discussed above. We suggest that asymptomatic patients with good VA and shallow SRF without hemorrhage, intraretinal fluid, or decreased vision can be observed closely. Indeed, many of our patients remained stable over several years without injections despite having shallow SRF and MNV by OCT-A.

In conclusion, OCT-A has substantially contributed to the improvement of our understanding in regard to the pathophysiology of retinal and choroidal vascular diseases including AMD and has helped us refine classification schemes over time. This could serve as a powerful screening and diagnostic tool in the clinical setting, leading to personalized and targeted management.

## Figures and Tables

**Table 1:** Types of neovascularization in AMD.

Types of MNV/CNV	Description	Fundus Photography	FA/ICGA	OCT	OCT-A
Type 1 (old term: occult CNV)	Neovascular complexes arise from the choriocapillaris into and within the sub-RPE space	Nonspecific signs: drusen, pigment mottling, RPE elevation, hemorrhages, hard exudates	FA: Lack of early hyperfluorescence or poorly defined: stippled hyperfluorescence over an area of elevated RPE within the first 2 min. Staining and/or leakage corresponding to RPE abnormalities in later phases/ICGA: presence of hyperfluorescent area (plaque)	Elevation of RPE by material with heterogeneous reflectivity often with overlying SRF (less common IRF).	New vessels below the level of the RPE
Type 2 (old term: classic CNV)	Neovascular complexes arise from the choroid, traverse BM and the RPE monolayer to proliferate in the subretinal space	Grayish subretinal lesion, retinal edema, hard exudates, subretinal and intraretinal hemorrhages	FA: Early hyperfluorescence; late leakage pooling in the subretinal space/ICGA: intense choroidal hyperfluorescence can impede CNV detection.	Disruption of inner/outer segment photoreceptor junction and intraretinal cysts	New vessels above the level of the RPE penetrating the retina
Type 3 (RAP)	Neovascular complexes arise either from the retinal circulation or from both circulations (retinal-choroidal anastomosis) and grow toward the outer retina	Focal intraretinal hemorrhages, dilated retinal vessels	FA: Focal and poorly defined hyperfluorescence associated with intraretinal staining in the early and late phase. Cystoid macular edema might be present	Sub-RPE CNV with intraretinal angiomatous change along with subretinal neovascularization and cystic change. Outer retinal disruption.	RAP lesions at the level of the avascular zone.
Mixed CNV-Type 1 and Type 2 variant	Varied presentation with findings of both Type 1 and Type 2 CNV	Nonspecific signs: drusen, pigment mottling, RPE elevation, hemorrhages, hard exudates	Findings of both Type 1 and Type 2 CNV	Findings of both Type 1 and Type 2 CNV	Findings of both Type 1 and Type 2 CNV
Polypoidal choroidal vasculopathy (Type 1 variant)	Branching vascular network and nodular vascular agglomerations (“polyps”)	RPE elevation, exudation,	FA: Stippled hyperfluorescence over an area of elevated RPE on late phase FA/ICGA: branching vascular network and aneurysmal dilations located at the outer edge of the lesion	“Polyps” located below RPE. Findings similar to type 1 CNV	Well-delineated branching vascular network. Aneurysmal dilations may not be seen (flow detection threshold not reached).
Quiescent CNV	Typical Type 1 CNV lacking exudation on repeated OCT for at least 6 months	Similar to type 1 CNV	FA: Late speckled hyperfluorescent lesions lacking well-demarcated borders. No late-phase leakage of undetermined source or pooling of dye in the subretinal space/In mid-late ICGA frames: visualization of hyperfluorescent “quiescent” CNV and plaques	Irregularly slightly elevated RPE without hyporeflective fluid accumulation in the intraretinal/subretinal space. Major axis in the horizontal plane, which is characterized by collections of moderately reflective material in the sub-RPE space and clear visualization of the hyperreflective Bruch’s membrane. Absence of CNV activity signs	During a period of stability or regression: capillaries and vessel loops no longer present. Remaining vessels are stiffer, thicker and less tortuous.
Subthreshold exudative CNV	Type 1 variant characterized by stable persistent or intermittent SRF	Drusen ± pigmentary changes, RPE elevation	FA: Stippled hyperfluorescence, late staining, no leakage/ICGA: absence of definite neovascular complexes and “hot spot,” rarely presence of plaque	Irregular RPE elevation with or without minimal SRF (wax and wane or persistent on FU)	Irregular vascular networks under the fovea or perifoveal areas

Abbreviations: AMD: age-related macular degeneration; CNV: choroidal neovascularization; MNV: macular neovascularization; RAP: retinal angiomatous proliferation; RPE: retinal pigment epithelium; BM: Bruch’s membrane; ICGA: indocyanine green angiography; SRF: subretinal fluid; IRF: intraretinal fluid; FA: fluorescein angiography; ICGA: indocyanine green angiography; OCT: optical coherence tomography; OCT-A: optical coherence tomography angiography; FU: follow-up.

## Data Availability

Not applicable.
